# Necrotizing enterocolitis after intravitreal bevacizumab in an infant with Incontinentia Pigmenti – a case report

**DOI:** 10.1186/s12887-019-1732-z

**Published:** 2019-10-15

**Authors:** S. Kunzmann, T. Ngyuen, A. Stahl, J. M. Walz, M. M. Nentwich, C. P. Speer, K. Ruf

**Affiliations:** 1 Department of Neonatology and Pediatric Intensive Care Medicine, Bürgerhospital Frankfurt am Main, Frankfurt, Germany; 2 Children’s Hospital, University of Würzburg, Würzburg, Germany; 3 Department of Ophthalmology, University Medicine Greifswald, Greifswald, Germany; 4 Eye Center, Medical Center, Faculty of Medicine, University of Freiburg, Freiburg, Germany; 5 European Foundation for the Care of Newborn Infants (EFCNI), Munich, Germany; 6 Eye Center, Medical Center, Faculty of Medicine, University of Würzburg, Würzburg, Germany

**Keywords:** Necrotizing enterocolitis, Incontinentia pigmenti, Bevacizumab, Retinopathy, VEGF

## Abstract

**Background:**

Incontinentia Pigmenti is a rare disease affecting multiple organs. Fifty of patients show affection of the eye with retinopathy and possible amaurosis being the worst outcome. Treatment has commonly been panretinal laser coagulation but intravitreal application of bevacizumab as VEGF-inhibitor has shown to effectively suppress retinal neovascularization.

**Case presentation:**

A six-week-old female infant with Incontinentia Pigmenti developed a foudroyant necrotizing enterocolitis shortly after intravitreal injection of bevazicumab due to a retinopathy with impending tractional detachment of the left eye. Since the onset of abdominal symptoms occurred immediately after the intravitreal application, a link between the two events seemed likely. Sequential analyses of the VEGF serum concentrations showed a massive suppression of endogenous VEGF with only a very slow recovery over weeks. Such a severe systemic adverse event has not been reported after intravitreal treatment with bevacizumab in an infant.

**Conclusion:**

This case report shows a relevant systemic uptake of bevacizumab after intravitreal application as suppressed VEGF levels show. There seems to be a connection between suppressed VEGF levels and the onset of necrotizing enterocolitis. Therefore, treatment with bevacizumab should be carefully considered and further research is needed to assess this drug’s safety profile.

## Background

Incontinentia pigmenti (IP) or Bloch-Sulzberger Syndrome is a rare X-linked dominant condition affecting neuroectodermal tissues [1, 2] and multiple organs. A mutation in the affected NEMO-gene (Xq28) can be found in most cases [3]. The “incontinence” is caused by a dislocation of melanin pigment from the basal cells of the epidermis to the upper dermis layer [4] and is characterized by skin lesions following the Blaschko lines [2]. Furthermore, alterations in teeth, hair and nails are common. In 30-50%, the central nervous system (CNS) with symptoms such as epilepsy, mental retardation, hemiparesis, spasticity and cerebellar ataxia is involved [1]. Up to 50% of the patients show eye abnormalities, among them retinopathy due to vascular occlusive changes [1, 5]. This retinopathy resembles retinopathy of prematurity (ROP) [5] with excessive pre-retinal angiogenesis which may result in tractional retinal detachment. This is believed to be an open door for treatment as performed in neonates with ROP [6]. Anti-VEGF agents such as bevacizumab gained importance in fields such as cancer treatment, intravitreal treatment of exudative age-related macular degeneration and ROP [7]. It may also be beneficial in cases of excessive pre-retinal neovascularization in IP as the data available shows [6, 8]. Little is known, though, about possible systemic adverse effects of anti-VEGF treatment in a pediatric population.

## Case presentation

**Fig. 1:**
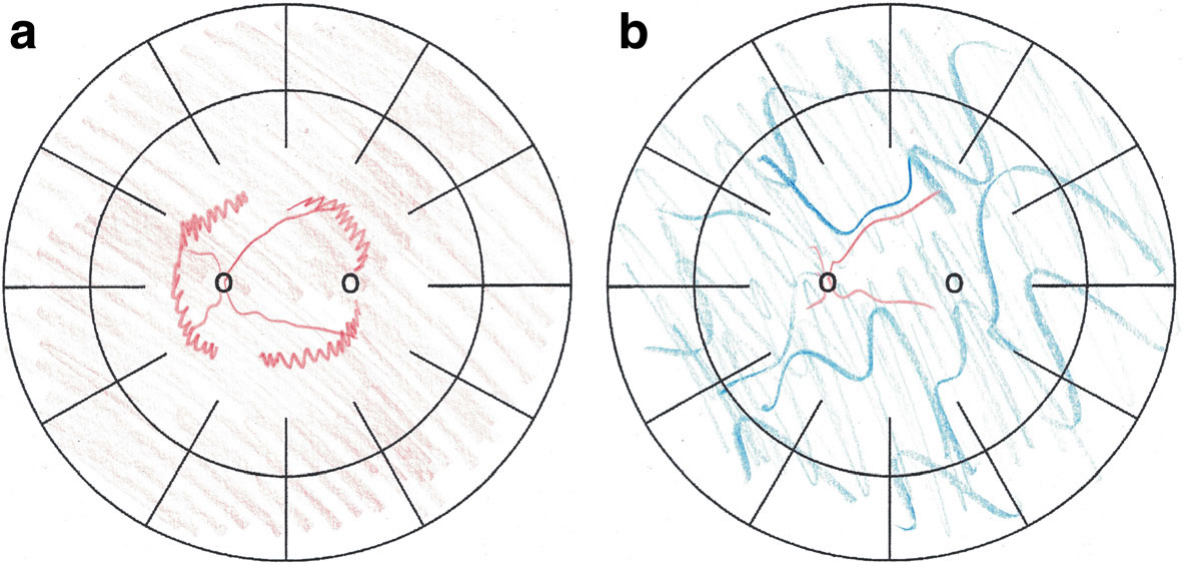
**a**: Retinal findings of the left eye: avascular areas and distinct extraretinal proliferation in zone1 at fundoscopy. **b**: Retinal findings of the left eye: retinal detachment due to progression of vitreo-retinal traction

The term-born female infant showed scaled skin lesions suspect for IP at the age of 3 weeks of life after an uneventful pre-, peri- and postnatal phase. The infant was formula fed and showed adequate weight gain. Eye examination showed vast avascular areas and extraretinal proliferations which were more pronounced on the left eye. These findings led to the diagnosis of IP, which in the meantime has been genetically confirmed (heterozygous mutation in the IKBKG gene). At the age of 6 weeks, the infant was referred to our hospital for further treatment. Neither on admission nor during the length of the hospital stay, CNS lesions were found in ultrasound examinations. Besides the mentioned scaled skin lesions, the child’s physical examination revealed no pathologies, abdominal palpation showed no tenderness or intra-abdominal mass and auscultation revealed normal bowel sounds. Due to the retinal findings and impending tractional retinal detachment with consecutive amaurosis, an *off-label* treatment with intravitreal bevacizumab (Avastin®) was performed in the left eye as bevacizumab is known to block neoangiogenesis and might stop a progressing retinopathy (see retinal findings in Fig. 1a). Based on data from studies in the treatment of ROP in preterm infants with bevacizumab, half of the adult dose (625 μg) was once applied intravitreally into the left eye [7, 9]. Subsequent retinal examinations, however, found aggravating neovascularization also of the right eye which made panretinal laser photocoagulation necessary 9 days after bevacizumab treatment of the left eye. Unfortunately, the application of bevacizumab could not prevent retinal detachment, which happened 8 days after application. This led to amaurosis of the left eye due to the extensive extraretinal proliferations already present at the initial presentation of the patient at our hospital. Post treatment fundus diagrams are attached as Fig. 1b. On the right eye, visual acuity at last presentation (December 2018) was 0.4 (tested with the Lea Hyvärinen optotypes), which is near-to-normal for children of this age.

**Fig. 2:**
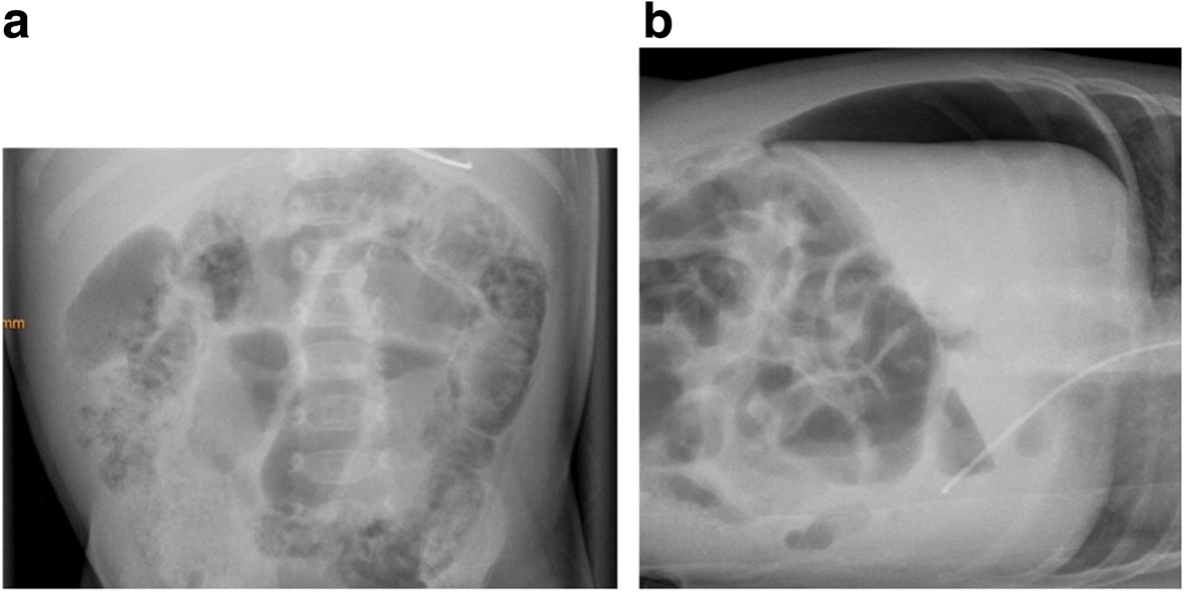
**a:** Supine abdominal X-ray 6 hours after intra-vitreal application of bevacizumab. Intramural gas (pneumotosis intestinalis) in the large bowel. **b:** Left lateral X-ray 8 days after intravitreal application of bevacizumab. Free air in the abdominal cavity, especially suprahepatic between liver, abdominal wall and right hemidiaphragm

Six hours post intravitreal anti-VEGF treatment, the infant presented with abdominal distension and bloody stools as signs of an acute necrotizing enterocolitis. Examination of the cardiovascular system showed no abnormalities X-ray confirmed a distinct pneumatosis intestinalis without signs for free abdominal air leaks (Fig. 2a) corresponding to Bell stage IIa. The infant was started on piperacillin, gentamicin and metronidazole as well as a fully parenteral diet; she was cardiorespiratory stable at all times without any respiratory support or the need for vasopressors. Eight days after the intravitreal treatment, the infant developed an acute abdomen with silent murmurs, visible dilated bowel loops and abdominal distension; the abdominal X-ray showed free abdominal air (Fig. 2b) corresponding to Bell stage IIIb. Besides a slightly altered coagulation (INR 1,7) and an elevated C-reactive protein 10 h after the onset of gastrointestinal symptoms (max. 6,5 mg/dl), no abnormalities were found in the blood tests, especially red and white blood cell and platelet counts were normal.

**Fig. 3:**
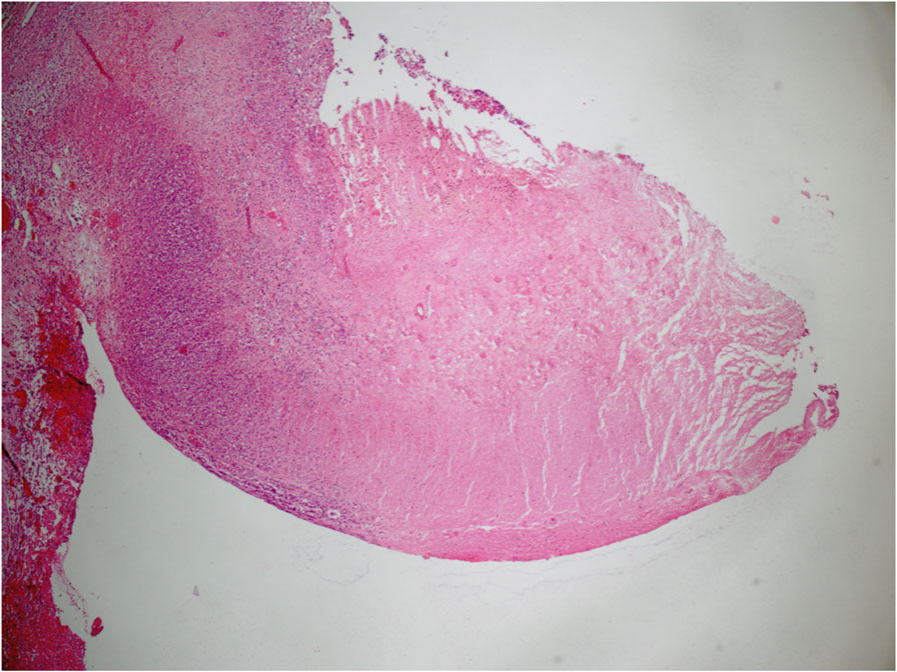
Necrotic bowel wall with inflammatory reaction. Histology was done at the time of intestinal perforation, which happened 8 days after the onset of NEC symptoms. HE stained, 40x magnification

In an immediate explorative laparoscopy, a pancolitis and a single focal perforation near the base of the appendix but no vascular malformations or malrotation were detected. The appendix as well as the perforated part of the coecum were removed. In total, 2.2 cm of intestine was resected. Pathological examination showed the typical picture of necrotizing enterocolitis (NEC) but no further gastrointestinal pathology (see Fig. 3). Postoperative enteral feeding was uneventfully completed after 14 days. One year after the event the child shows normal feeding and an adequate weight gain.

**Fig. 4:**
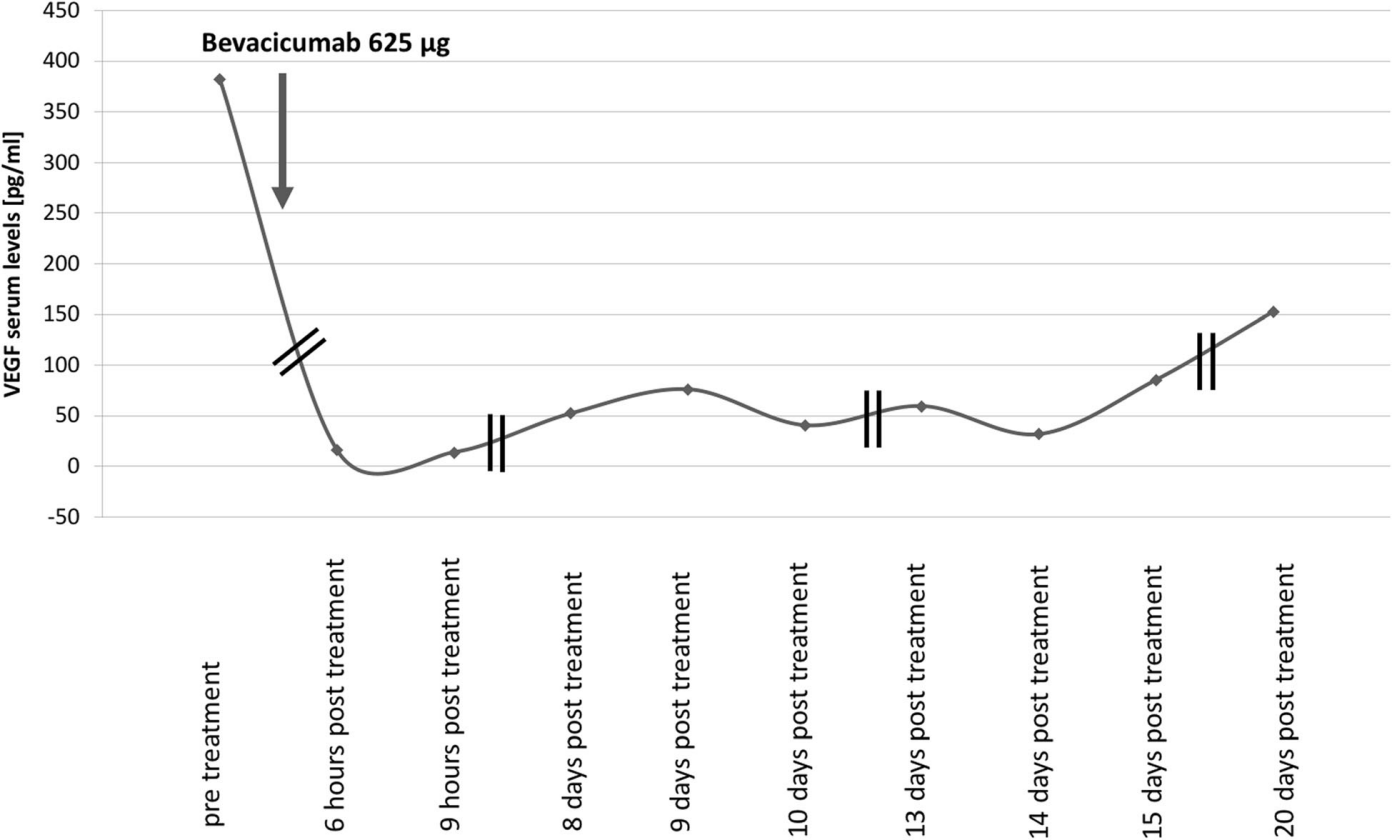
Serum VEGF levels before and after bevacizumab showing a drastic suppression of VEGF serum concentration and a slow recovery that has not reached the pre-application level after almost 3 weeks

The short onset of gastrointestinal symptoms after intravitreal anti-VEGF application suggested a possible link between the two. To investigate a possible adverse drug effect, we compared serum VEGF levels before and after bevacizumab application using ELISA (R&D Cat No. DVE00) following the manufacturer’s instructions. Serum samples from 1 day before the injection of bevacizumab, 6 and 9 h as well as 7, 8, 9, 12, 14, and 19 days after the injection were available for analysis. Figure 4 shows a drastic decrease of serum VEGF concentrations just hours after intravitreal application of bevacizumab and only a slow recovery: 3 weeks after application the concentration reached only a third of the baseline VEGF concentration. The current standard dose of 625 μg bevacizumab used for ROP treatment in much younger infants was thus sufficient to fully suppress endogenous free serum VEGF for more than 3 weeks in our patient.

## Discussion and conclusions

This is the first report of an infant with pathognomonic skin lesions and ophthalmological complications of IP that developed a NEC shortly after intravitreal application of bevacizumab. As NEC has not been reported in IP and is rarely seen in full-term infants and the onset of symptoms was time-related to intravitreal anti-VEGF application, we hypothesized a possible link between the single intravitreal application of bevacizumab and the development of the NEC/pancolitis as a systemic adverse event of intravitreal application of bevacizumab.

There are numerous studies concerning intravenous application of bevacizumab and other anti-VEGF agents mainly used in cancer treatment [10, 11], which are considered safe and well tolerated in adult patients. Only few authors report serious adverse events including hemorrhage, arterial thromboembolic events and gastrointestinal infarction [12]. Intravitreal application of anti-VEGF agents is common in the treatment of neovascular age-related macular degeneration, diabetic macular edema and other adult retinal disorders. In these patients, reported adverse events after intravitreal anti-VEGF treatment are rare [13, 14]. Only few reports of gastrointestinal complications such as an acute abdomen after intravitreal application of anti-VEGF drugs have been published [15, 16].

To our knowledge, this is the first report of an infant with a time-related necrotizing enterocolitis following intravitreal application of bevacizumab. Although some authors report on decreases of VEGF serum levels after the application of intravitreal bevacizumab or ranibizumab in infants with ROP [17, 18], data on severe systemic side-effects after intravitreal anti-VEGF application in infants is scarce. Lately, concerns have been raised regarding the systemic absorption of anti-VEGF antibody after intravitreal injection in preterm infants and its effect on developing tissues including the CNS. One study, where preterm infants were treated with intravitreal bevacizumab, postulated a higher rate of severe neurodevelopmental disabilities in comparison to laser treatment [19], a finding that could not be confirmed by another study [20]. Recently, the results of a randomized, prospective, double-blind multi-center trial investigating the safety and efficacy of different doses of ranibizumab (another anti-VEGF antibody) in the therapy of ROP were published and showed no systematical alteration of VEGF plasma levels, which points towards a limited systemic drug exposure after intravitreal therapy with ranibizuma b[21]..

The patient reported here has IP as underlying disease. As to the pathogenesis of many alterations in IP, inflammatory and vascular malfunction are believed to be possible causes of uveitis, retinal and cerebral bleeding, mental retardation or ataxia [1, 2]. A gastric involvement has not been reported so far. Similar to IP, inflammatory and vascular malfunction are involved in the pathogenesis of NEC [22]. NF-kB signaling, which is disturbed by NEMO mutations in IP patients [3], is also found in the gut epithelium. NEMO knock-out mice have a primary gut epithelial defect resulting in an impaired integrity and antimicrobial defense of the gut epithelium leading to intestinal inflammation [23]. Furthermore, NF-kB signaling in intestinal epithelial cells emerges as a crucial factor for maintaining homeostasis between commensal microflora and host immunity in the gastrointestinal tract, and represents a critical determinant for prevention of gut inflammation [23] which is postulated to be dysregulated in the pathogenesis of NEC [22]. Thus, both pathologies in our reported patient, the IP and the NEC are somehow related to a disturbed NF-kB signaling. Whether the NEC has been triggered through an already disturbed NF-kB pathway in our patient remains speculation.

No recommendations for applying intravitreal anti-VEGF drugs in preterm infants who sustained intestinal injury, e.g. a necrotizing enterocolitis, have been made so far as no information on possible adverse effects of such substances to an immature and possibly already damaged intestinal system is available. In a mouse model and in human infants with NEC, VEGF in the intestines is decreased [24]. Furthermore, lack of the VEGF receptor 2 causes maldevelopment of the intestinal microvasculature and facilitates NEC in mice [25] and genetic polymorphisms for VEGF is an independent risk factor for NEC [26]. Vice versa VEGF overexpression seems to be a promising approach in the management of NEC, as demonstrated in a rat model [27].

This report is the first to describe a link between intravitreal application of anti-VEGF treatment and time-related development of NEC in an infant with IP. Since drastically reduced VEGF serum concentrations were found, a relation of the intravitreal application of bevacizumab and the development of an ischemic intestinal injury seems at least possible, however, based on a single patient, a causative relationship remains speculation. Therefore, anti-VEGF therapy in patients with already altered intestinal epithelium or - as in our case - with a possibly higher risk of NEC due to a changed NF-kB pathway should be very well considered. Ranibizumab, having a shorter half-life than becacizumab, may be the agent of choice due to a reduced risk of systemic uptake in such infants.

## Data Availability

Not applicable.

## Abbreviations

CNS: central nervous system
IP: incontinentia pigmenti
NEC: necrotizing enterocolitis
ROP: retinopathy of prematurity
VEGF: vascular endothelial growth factor
